# Virological pattern of hepatitis B infection in an HIV-positive man with fatal fulminant hepatitis B: a case report

**DOI:** 10.1186/1752-1947-3-110

**Published:** 2009-11-09

**Authors:** Sabrina Bagaglio, Luca Albarello, Priscilla Biswas, Caterina Uberti-Foppa, Claudio Fortis, Giulia Morsica

**Affiliations:** 1 Infectious Diseases Department, San Raffaele, Scientific Institute, Via Stamira d'Ancona, Milano 20127, Italy; 2 Division of Pathology, San Raffaele, Scientific Institute, Via Olgettina, Milano 20132, Italy

## Abstract

**Introduction:**

There seem to be no published data concerning the clinical impact of populations of hepatitis B virus (HBV) in the hepatic and extrahepatic compartments of HIV-infected people with severe acute hepatitis.

**Case presentation:**

A 26-year-old Caucasian man presenting to our hospital with clinical symptoms suggesting acute hepatitis was found to have an acute hepatitis B profile upon admission. He developed fatal fulminant hepatitis and was found to be heavily immunocompromised due to HIV-1 infection. He had a high plasma HBV and HIV load, and analysis of the partial pre-S1/pre-S2 domain showed the presence of mixed infection with D and F genotypes. Analysis of the point mutations within this region revealed the presence of HBV strains with amino acid substitutions at the immunodominant epitopes involved in B or T cell recognition. A homogeneous population of a pre-core mutant strain harbouring the A1896G and A1899G affecting HBeAg expression was invariably found in the liver tissue, plasma and peripheral blood mononuclear cells despite active HBeAg secretion; it was the dominant strain in the liver only, and was characterised by the presence of two point mutations in the direct repeat 1 domain involved in HBV replication activity. Taken together, these mutations are indicative of a highly replicative virus capable of evading immune responses.

**Conclusion:**

This case report provides clinical evidence of a possible association between the rapid spread of highly replicative escape mutants and the development of fulminant hepatitis in a heavily immunocompromised patient. Virological surveillance of severe acute hepatitis B may be important in establishing an early treatment strategy involving antiviral drugs capable of preventing liver failure, especially in individuals for whom liver transplantation is not accepted as a standard indication.

## Introduction

Various viral mutations have been implicated in the etiology and pathogenesis of fulminant hepatitis B (FHB) and mutations within the pre-core (preC) region of hepatitis B virus (HBV) have been detected in some cases [1]. The preC region of the HBV genome is a short open reading frame (nucleotide 1814-1901) preceding the core gene that contains the epsilon signal sequence for viral encapsidation, which is essential for the start of HBV DNA synthesis [2]. Mutations in this domain could therefore hamper the mechanism of viral replication. The most frequently encountered point mutation involving the lower stem of the epsilon structure is the A instead of G mutation at position 1896 that induces a stop codon in the preC gene, affects HBeAg expression and has been associated with a severe course of acute hepatitis [3].

An 11-base pair sequence, direct repeat-1 (DR-1), which starts at nucleotide 1824 of the preC region is directly repeated near the extremity of the viral plus strand DNA. It is remarkably well-conserved among different HBV isolates, and the fact that it is necessary for the formation of plus strand and relaxed circular (RC)-DNA [4] means that it plays a pivotal role in HBV replication.

Eight genotypes (A-H) have been identified on the basis of their >8% sequence divergence in the entire genome. Their distribution varies from country to country, with genotype D being prevalent in the Mediterranean basin.

Various lines of evidence suggest that HBV may infect peripheral blood mononuclear cells (PBMCs) [5], which may therefore represent an extrahepatic site of viral persistence and play a crucial role in exacerbating liver disease in chronic HBsAg carriers.

We investigated the hepatic and extrahepatic patterns of HBV infection in a patient who was also infected with HIV and who was participating in a prospective study of acute hepatitis B, which fatally evolved into FHB.

## Case presentation

A 26-year-old Caucasian man was referred to our hospital with jaundice and symptoms of general fatigue and anorexia. He denied any known risk factors for potential exposure to HBV or HIV, including no history of intravenous drug use, surgery, tattoos or piercing. The laboratory findings upon admission showed a platelet count of 139 × 10^9^/litre and a prothrombin time of 34.4 seconds.

The patient had very few CD4^+ ^(12 cells/μl) and CD8^+ ^(323 cells/μl) lymphocytes and a CD4^+^/CD8^+ ^ratio of 0.04. Blood chemistry tests showed total and direct bilirubin levels of 1.56 mg/dl and 0.60 mg/dl, respectively, aspartate aminotransferase (AST) 2037 IU/litre, alanine aminotransferase (ALT) 2317 IU/litre, lactate dehydrogenase (LDH) 1395 IU/litre and alkaline phosphatase (AP) 180 IU/litre. Abdominal sonography revealed an enlarged liver with a dishomogeneous structure and normal biliary tree. The patient had an acute hepatitis B profile, being positive for HBsAg and HBeAg, weakly positive for anti-HBc IgM and anti-HBc IgG, and negative for anti-HBeAb. He was negative for the markers of acute hepatitis A, hepatitis delta virus, cytomegalovirus and Epstein-Barr virus, as well as for anti-HCVAb and HCV RNA, but positive for anti-HIVAb. Western blotting confirmed a serological profile of chronic HIV infection; as the patient did not know he was seropositive for HIV, this was his initial diagnosis with HIV. His sexual partner was tested and found negative for both HBV and HIV infection.

Table 1 summarises the sequential serological markers of HBV/HIV co-infection and the patient's immunological status.

**Table 1:** Characteristics of HBV/HIV co-infection in an HIV-positive man with FHB.

	Admission	Before death
HBsAg	+++	+++

HBsAg	+++	+

HBsAb	neg	Neg

HBsAb	neg	Neg

HBcAb (IgM)	+/- ^a^	+++

HBcAb (IgG)	neg	+

HIV-1 Ab	+	ND

		
Antigag Ab	pos	ND

Antipol Ab	pos	ND

Anti-env Ab	pos	ND

CD4^+ ^cell count		

n/μl	12	11

CD8^+ ^cell count		

n/μl	323	310

HBV-DNA copies/ml	1.5 × 10^9^	5 × 10^8^

HIV-RNA copies/ml	3.6 × 10^5^	ND

^a^+/- = weakly positive; ND = not done.

Over the following week, total bilirubin rapidly increased to 12 mg/dl, ALT to 3378 IU/l, and AST to 4235 IU/l. His level of consciousness rapidly deteriorated, coagulation time became prolonged, and he was diagnosed as having FHB.

Treatment with lamivudine 200 mg/day was started on day 12 after hospital admission, but was ineffective and he died of liver failure 3 days later. The autopsy findings showed a slightly reduced liver volume and consistency. Liver histology was scarcely valuable because of massive necrosis and severe autolithic phenomena.

Plasma HBV DNA was quantified using a real-time polymerase chain reaction (PCR) assay according to the manufacturer's instructions (RealArt HBV™ PCR kit; QIAGEN Diagnostics GmbH, Hamburg, Germany; lower sensitivity limit: 60 IU/ml = 312 copies/ml). HIV load was quantified using a branch-DNA assay (Versant HIV-RNA 3.0, Bayer SpA, Milan, Italy). PBMCs (10^6 ^cells) were isolated by means of Ficoll-Hypaque density-gradient centrifugation and resuspended in 10 ml RPMI 1640 medium (Lonza-BioWhittaker Verviers, Belgium). Upon admission, the patient had high plasma levels of HBV DNA and HIV RNA, and a high HBV DNA load was also found 1 day before his death (day 14 after hospital admission, Table 1).

HBV DNA was extracted from formalin-fixed paraffin-embedded liver tissue sections (10 μm), PBMCs and from 200 μl of plasma by means of the QIAamp DNA Mini-kit (Qiagen SpA, Milan, Italy) following the manufacturer's instructions.

The HBV genotype was determined by means of the amplification (hemi-nested PCR) of the partial pre-S1/pre-S2 region, followed by the sequence analysis of 30 clones. We decided to characterize the HBV genotype by performing the analysis of clones because the concomitant infection of the same host with different HBV genotypes is accurately determined by using this molecular approach.

The preC/C viral population was investigated by sequencing 18-20 clones propagated from liver, PBMCs and plasma for a total of 87 clones, in order to explore the compartmentalisation of the preC mutated strains and their possible implication in the pathogenesis of the FHB.

**Figure 1:**
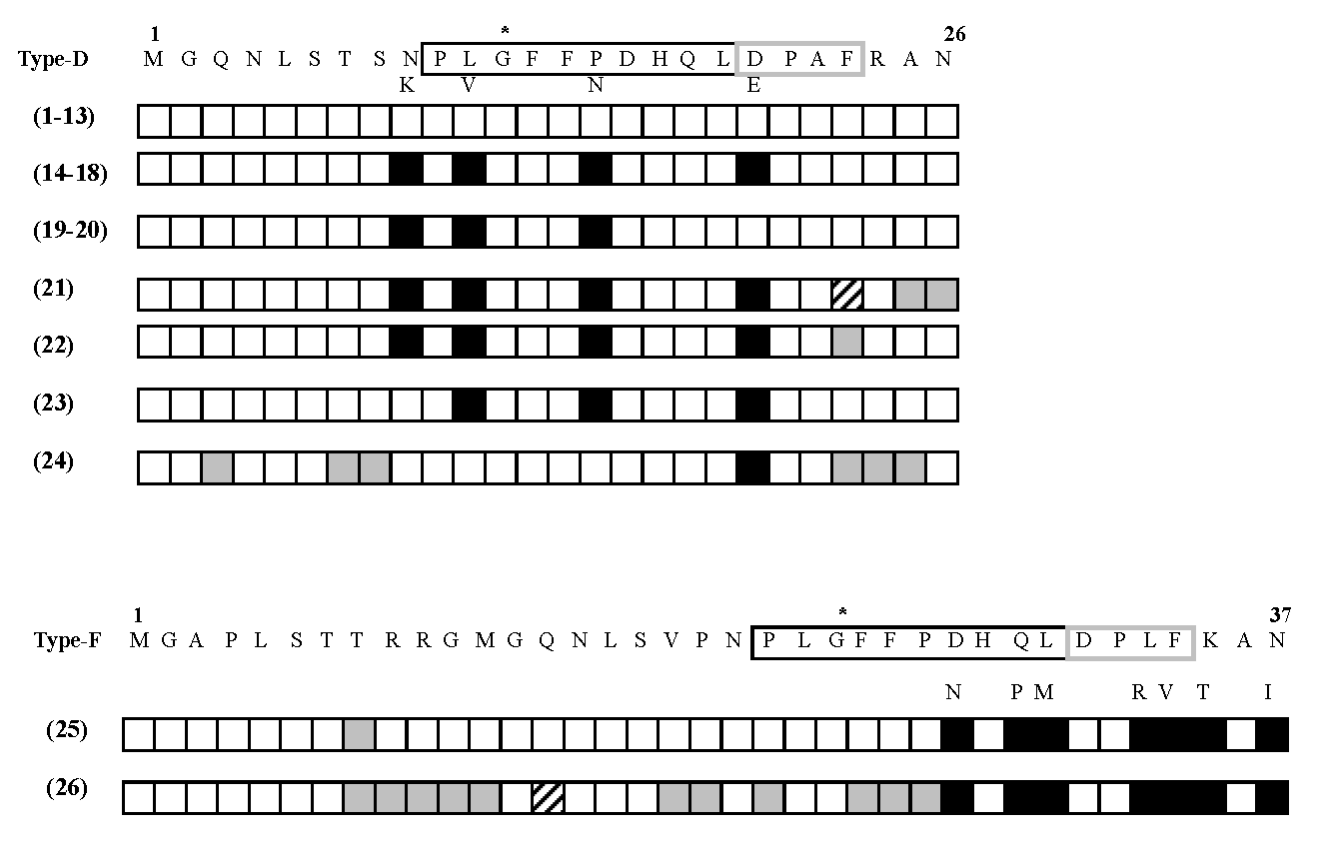
**Analysis of the pre-S1 amino acid sequences of 26 clones derived from the plasma sample**. A minor population clustering with genotype D showed glutamic acid/aspartic acid (E/D) amino acid substitution within the immunodominant epitope responsible for the hepatocyte binding site, and lysine/asparagine (K/N), valine/leucine (V/L), asparagine/proline (N/P) aa changes in the epitopes recognized by B and T lymphocytes. The alignment of clones 1-24 clustering with type D, and clones 25 and 26 clustering with genotype F, was made on the basis of the sequence of the HBV prototype. The numbers in parentheses refer to the total number of clones with identical amino acid sequences. The empty box indicates identical amino acid; the black box indicates the amino acid substitution (found in at least nine genotype D clones and the two genotype F clones; the grey box indicates a randomly detected amino acid mutation; and the striped box indicates an aa deletion. The pre-S1 epitopes responsible for immune response at T-cell level or thought to contain the hepatocyte binding site are respectively boxed in black and grey; the asterisk indicates the start of the sequence of the pre-S epitope that elicits the B cell immune response.

A sequence analysis of 26 molecular clones propagated from plasma revealed mixed HBV D/F genotype infection: 24 clones clustered with genotype D and two with genotype F. Detailed sequence analysis showed that the dominant D strain did not show any amino acid (aa) changes, whereas a minor population clustering with genotype D showed aa substitution within the immunodominant epitope responsible for the hepatocyte binding site, and aa changes in the epitopes recognized by B and T lymphocytes (Figure 1). The two clones belonging to genotype F showed aa changes in the immunodominant B and T epitopes, as well as within the hepatocyte binding site (Figure 1).

**Figure 2:**
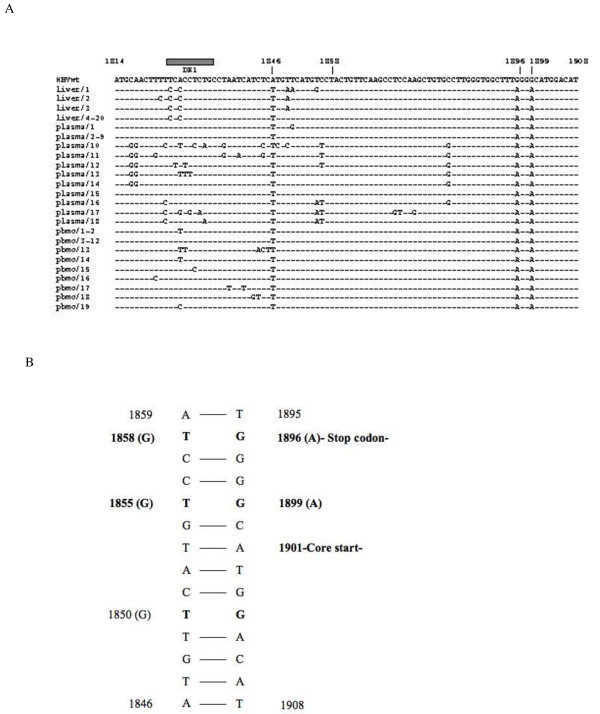
**Alignment of the pre-core clone sequences propagated in different compartments and schematic representation of the secondary structure of the HBV pre-genome encapsidation signal**. A) Alignment of the pre-core clone sequences propagated in different compartments. The alignment was made on the basis of the wild-type sequence of the pre-genome encapsidation signal. The dashes (-) represent the nucleotides that are identical to the wild-type sequence. B) Schematic representation of the secondary structure of the HBV pre-genome encapsidation signal: base pairing of the lower stem. The main nucleotide substitutions that better stabilise the epsilon structure detected in the clones derived from liver tissue, peripheral blood mononuclear cells and plasma are shown in parentheses.

A sequence analysis of 20 independent clones propagated from liver revealed the presence of a homogeneous preC mutated population showing the A1896G stop codon and the A1899G substitution, which stabilised the epsilon structure better, thus avoiding a possible decrease in viral replication; only three clones had a divergent sequence with additional nucleotide point mutations. Genetically related preC mutants harbouring the A1896G substitution, which prevents the formation of the preC protein, were found in 19 clones propagated from PBMCs. The point mutation A1899G, which further stabilises the structure of the epsilon region by pairing with T1855, was invariably detected in PBMCs and plasma (Figure 2). Some of the clones propagated from PBMCs and plasma were identical, but genetically divergent from those detected in liver tissue (Figure 2).

Notably, all of the clones from the different compartments had the preC stop codon mutant and the point mutation A1899G.

Interestingly, a mutant strain with T/C and A/C substitutions at the 5' of the direct repeat (5'-DR1) region, was invariably found in the clones propagated from the liver compartment, but was not detected in those propagated from PBMCs or plasma (Figure 2).

## Discussion

HBV is a non-cytopathic virus in which virus-specific immune responses are thought to play a central role not only in mediating viral control but also in initiating liver injury. However, FH occurred in our patient with HIV despite clinical evidence of his severely immunocompromised status.

To the best of our knowledge, this is the first report of mixed HBV D/F genotype infection in plasma, and preC stop codon dominance in different compartments. HBV genotype F was originally isolated in the Amerindian populations of the Americas and is extremely unusual in Europe [6]. However, our finding is in line with those published in single reports from Europe and Japan [7,8] describing its presence in immunocompetent subjects and patients infected with HIV.

It is worth noting that we detected aa mutations within epitopes of the pre-S1 domain, which are important for attaching the virus to the target cells and for stimulating B and T cell immune responses [9] in some clones clustering with genotype D and the two clones clustering with genotype F. We hypothesise that these specific aa substitutions may modify the binding of the virus to its target cells with a possible effect on virus entry, and may induce a conformational change in B and T cell epitopes that favours the escape of mutants from the specific immune response. However, these hypotheses are formed on the basis of the genetic characteristics of the virus from this one patient and need to be confirmed by experimental propagation in animal models or cell lines.

The serological data indicated the presence of HBeAg despite the detection of a homogeneous population of preC stop codon mutants, and therefore it is possible that it was produced by a minor viral population clustering within genotype F [10].

Two nucleotide substitutions in the 5'-DR1 region, which is an essential *cis *element for hepadnaviral reverse transcriptase and immediately precedes the 5'epsilon region, were detected in all of the clones derived from the liver compartment. It has been shown that base-pairing interactions in minus strand DNA are critical for efficient primer translocation and RC formation. As RC-DNA genomes may have a competitive advantage over duplex linear (DL)-DNA genomes in initiating infection [11], our finding of nucleotide mutations in DR1 may indicate reduced complementarity between the RNA primer and DR2, thus affecting primer translocation, RC formation and, consequently, viral DNA synthesis. However, there are no conclusive results concerning the significance of DL-DNA or RC-DNA production on the virus life cycle.

Several studies have shown that HBV DNA is present in PBMCs and that HBV may replicate in these cells. The infection of PBMCs by HBV could interfere with the host's immune defense against the virus and may support the establishment of HBV persistence in acute hepatitis B, or in HBV carriers after liver transplantation, with important clinical consequences [12,13].

In our case report, the presence of HBV DNA in PBMCs was shown, and sequence analysis of the preC region identified HBV strains in PBMCs and plasma that were not closely related to each other. The most likely explanation for this is that different plasma and PBMC compartments may have host biological conditions that differ from those of the liver, thus leading to the dominance of a well-adapted variant in these sites of replication.

Finally, it is worth knowing that our FHB patient had a high HBV DNA titre at baseline one day before he died.

A recent report [14] indicates that treatment for severe acute hepatitis should be recommended in order to reduce the risk of progression to FH and the need of organ liver transplantation.

Unfortunately, treatment with lamivudine was not started until 12 days after admission, by which time the clinical condition of this patient had greatly deteriorated.

## Conclusion

This is the first report describing a case of acute hepatitis B with a fulminant course in a heavily compromised HIV-positive patient showing the dominance of HBV genotype D over genotype F in plasma, and the selection of preC mutant strains with different genetic characteristics in hepatic and extrahepatic sites. It would be interesting to determine whether this virological pattern may be specifically related to severe FH by extending this analysis to a larger number of patients with acute severe hepatitis B as this could add important information for its management. In selected cases, early treatment with antiviral drugs could prevent the need for liver transplantation or prevent a fatal outcome.

## Abbreviations

AA: amino acids; ALT: alanine aminotransferase; AP: alkaline phosphatase; AST: aspartate aminotransferase; D: aspartic acid; DL-DNA: duplex linear DNA; DR-1: direct repeat 1; DR-2: direct repeat 2; E: glutamic acid; FHB: fulminant hepatitis B; K: lysine; L: leucine; LDH: lactate dehydrogenase; N: asparagine; P: proline; PBMC: peripheral blood mononuclear cells; RC-DNA: relaxed circular DNA; V: valine.

## Competing interests

The authors declare that they have no competing interests.

## Authors' contributions

SB performed, analysed and interpreted the experimental studies, and made a major contribution to the writing of the manuscript. LA performed the histological examination of the liver and actively contributed to writing the final version of the manuscript. PB gave technical help and quantified HBV in sequential specimens. CU and CF collected, analysed and interpreted the clinical data regarding the acute liver failure. GM was responsible for designing the study in terms of the clinical and virological data analysis and made a major contribution to writing the manuscript.

## Consent

Written informed consent could not be obtained in this case as the patient is dead and his next of kin could not be traced. However, we believe that this case report contains a clinical lesson that cannot be effectively conveyed in any other way. We do not expect the next of kin (or any reasonable person) to object to publication as the patient cannot be identified.
